# Supplementary material for: Learning curve and surgical outcome of robotic assisted colorectal surgery with ERAS program
Source: Sci Rep. 2022 Nov 29;12:20566. doi: 10.1038/s41598-022-24665-w (PMC9709162; doi:10.1038/s41598-022-24665-w)
Supplement: Supplementary file 2 — Supplementary Information 2. [file 41598_2022_24665_MOESM2_ESM.zip › RAL Raw data-Ñ[▒K/100/Patho_ñw╝╨░O▒Kñσ.pdf]

臺中榮民總醫院 臺中市臺灣大道四段1650號 病理部活體組織切片 臨床醫學實驗及病理診斷中心 檢驗(查)報告

[redacted]

Pathologic diagnosis:
1. Ascending colon and terminal ileum, right hemicolectomy --- a. Adenocarcinoma, moderately differentiated, involving whole layer of terminal ileum and mesocolic soft tissue. See Comment. The ileocecal valve, bilateral cut ends and mesocolic lymph nodes (0/13) are free of tumor. b. Foreign body type granuloma. c. Inverted appendix.
2. Abdominal wall, resection --- Metastatic adenocarcinoma, moderately differentiated, in soft tissue and skeletal muscle.

Ancillary study for diagnosis:
IHC stains for PMS2 and MSH6 are done on section E. All above markers are preserved.

Gross description:
The specimen consists of 1) a segment of colon, 12 cm in length, and 7 cm in proximal circumference, 6.5 cm in distal cut end circumference, and a segment of terminal ileum, 6 cm in length, and 3.5 cm in cut end circumference. An polypiod lesion, 1.3x0.8x0.6 cm, arising from the cecal mucosa, is noted, 1 cm to the ileocecal valve. A gray tan firm annular elevated and centrally ulcerative tumor, 2.5x2.2 cm, is noted in the ileum, 2.3 cm to the proximal cut end. On cross section, the tumor invaded to the muscle layer with 3 mm to the radial margin grossly. A firm nodule, 1.6x1.5x1.2 cm, is noted in the mesenteric soft tissue. The ileocecal valve, and both cut ends are free of tumor. 2) two pieces of tan brown soft tissue, up to 5.2x4.5x2.5 cm. On cut, there is a whitish firm tumor ntoed in the specimen, very close to the resected margin. Representative part for section: A) proximal cut end B) distal cut end C) ileocecal valve D) polyp (inverted appendix) E-F) tumor with radial margin G) tumor with ileum H) ileum J) colon K) mesenteric nodule L-P) specimen 2 Q-R) lymph nodes.

Comment:
Combined with the clinical history and clinical presentation, the possibility of metastatic adenocarcinoma from colorectal origin could be compatible.

Note:
1. The interpretation of MMR panel and recommended studies are as below: (1) No loss of nuclear expression of MMR proteins: low probability of microsatellite instability-high (MSI-H) . (2) Loss of nuclear expression of MLH1 and PMS2: testing for methylation of the MLH1 promoter and/or mutation of BRAF is indicated (the presence of a BRAF V600E mutation and/or MLH1 methylation suggests that the tumor is sporadic and germline evaluation is probably not indicated; absence of both MLH1 methylation and of BRAF V600E mutation suggests the possibility of Lynch syndrome, and sequencing and/or large deletion/duplication testing of germline MLH1 may be indicated). (3) Loss of nuclear expression of MSH2 and MSH6: high probability of Lynch syndrome (sequencing and/or large deletion/duplication testing of germline MSH2 may be indicated, and, if negative, sequencing and/or large deletion/duplication testing of germline MSH6 may be indicated). (4) Loss of nuclear expression of MSH6 only: high probability of Lynch syndrome (sequencing and/or large deletion/duplication testing of germline MSH6 may be indicated). (5) Loss of nuclear expression of PMS2 only: high probability of Lynch syndrome (sequencing and/or large deletion/duplication testing of germline PMS2 may be indicated). (6) Please note: There are exceptions to the above IHC interpretations. These results should not be considered in isolation, and clinical correlation with genetic counseling is recommended to assess the need for germline testing.
2. This case has been peer reviewed by two doctors.

#T-58604_2 #M-81406_2 2 1105 CW000F
#T-03480_2 #M-81406_2 2 1105 000000
#T-C4560_2 #M-09183_2 0 1105 000000
#T-59200_2 #M-31315_2 0 1105 000000

已電子簽章

敬告：本單僅供醫師參考使用，不具任何申請效力！！
請務必妥為保管，否不慎遺失需自負個人資料外洩責任！
